# Private companies and community collaboration: Towards building disaster resilience in Diepsloot, Johannesburg, South Africa

**DOI:** 10.4102/jamba.v13i1.1003

**Published:** 2021-11-25

**Authors:** Modiegi Bopape, Livhuwani D. Nemakonde, Kristel Fourie

**Affiliations:** 1 African Centre for Disaster Studies, Faculty of Natural and Agricultural Sciences, North-West University, Potchefstroom, South Africa; 2 Proactive Health Solutions, Johannesburg, South Africa

**Keywords:** collaboration, disaster risk reduction, resilience, vulnerability, South Africa, community resilience, private sector

## Abstract

The responsibility for building community resilience cannot and should not rest with the public sector alone. It requires all sectors to collaborate for the benefit of the entire community. Specifically, it is important for private sector organisations to participate in building community resilience because they have vested interest in the area because of their physical assets, suppliers, customers and corporate value of social responsibility. This article explores collaboration between private companies and community of Diepsloot, Johannesburg, South Africa, to build disaster resilience in the community. The study applied qualitative research methods. Data were collected through focus group interviews with the community of Diepsloot and semi-structured individual interviews with representatives of private companies operating in the vicinity of Diepsloot. A total of 55 respondents participated in the study. Respondents included five corporate social responsibility (CSR) managers from private companies and 50 community members. The findings of the study showed that private companies are involved in addressing socio-economic challenges in Diepsloot. Addressing such challenges contributes a great deal to reducing exposure to hazards and the vulnerability factors to disasters, thereby contributing to building resilience. Whereas some respondents preferred the private companies to work with the communities directly, the study recommends the use of community structures such as Community Based Organisations (CBOs) when private companies engage in community initiatives. The article contributes to better understanding of the private sector’s contribution to build community resilience.

## Introduction

In South Africa (SA), the involvement of the private sector in efforts to build community resilience is still minimal. This is despite the disaster risk management (DRM) legislation in SA highlighting the need for involvement of private companies in disaster risk reduction (DRR) and building resilience (South African Government 2002). According to Van Niekerk, Ndlovu and Chipangura (2015), there is a need for the SA Government to actively engage the private sector in disaster risk efforts, such as policy development, risk mitigation, risk response and building resilience. The Disaster Management Framework of South Africa of 2005 highlights the need for the SA Government to facilitate the involvement of private sector, non-governmental organisations (NGOs), traditional leaders, technical experts, volunteers and the community. The aim is to form collaborative efforts in DRM and DRR, especially strengthening disaster resilience in communities (South African Government 2002).

The United Nations (UN) (2016) defines DRM as the application of DRR policies and strategies to prevent new disaster risk, reduce existing disaster risk and manage residual risk, contributing to the strengthening of resilience and reduction in disaster losses. Moreover, UN (2016) states that DRR is aimed at preventing new and reducing existing disaster risk and managing residual risk, all of which contribute to strengthening resilience and therefore achievement of sustainable development. In this regard, DRR is the policy objective of DRM, and its goals and objectives are defined in DRR strategies and plans (UN 2016). However, in most cases the public sector is constrained by fiscal deficits to implement DRR strategies and plans. This necessitates the involvement of other stakeholders such as the private sector and the civil society.

Whereas there has been much advocacy on the need for private sector involvement in DRR (see next section), there is little evidence of private sector involvement in DRR and building disaster resilience in SA. There is a dearth of studies to demonstrate the role that private companies are playing to reduce the risk of disasters in the country. This study investigates collaboration between private companies in the vicinity and community of Diepsloot, Johannesburg, towards building disaster resilience. In this regard, the private sector is considered to comprise a mix of multinational companies, national companies, small-medium enterprises and the informal economy. This study focuses on small-medium enterprises. In the following sections, we briefly provide a literature review on the role of private companies in DRR, briefly introduce the research context and methods, present and discuss the findings before conclusions are drawn.

## Collaboration for building community resilience: Conceptual justification

Communities bear the brunt of the increasing risks and impacts of disasters. Disasters and their impacts put communities at risk of losing their lives and livelihoods, injury, damage to property and infrastructure they depend on, and economic disruptions. Disasters occur because of exposure to hazards, conditions of the vulnerability of the exposed elements and insufficient capacity to cope with hazardous events. Together with resilience, vulnerability represents approaches to understanding the responses of systems and actors to hazards (eds. Fuchs & Thaler 2018). However, it must be noted that resilience and vulnerability are not the opposite but are rather related conceptually (Cutter 2016). To support this notion, Cutter (2016) states that communities and social groups contained within the concepts of vulnerability and resilience can be highly vulnerable, yet that does not mean they lack resilience.

Vulnerability is broadly defined as the potential for loss. It includes elements of exposure (people, places, infrastructure at risk from hazards), sensitivity (the degree to which people, places and infrastructure are harmed) and coping (the skills, resources and opportunities of people and places to survive, absorb the impacts and manage the adverse outcomes) (eds. Fuchs & Thaler 2018). According to Gil-Rivas and Kilmer (2016), disaster vulnerability is best understood to be a result of the combined effects of the characteristics of an individual, group or community as well as the social, economic and political factors that influence their capacity to anticipate, cope and recover from a disaster. Boin and Hart (2006) share similar views and point out that the causes of vulnerability are inherent in the social and economic systems made up of ethnicity, race and socio-economic status. McEntire, Crocker and Peters (2010) argue that these variables are significant in creating disaster risk and they correlate with an increase in vulnerability to hazards and that they often result in disasters. Identifying factors that influence vulnerability to hazardous events provides a mechanism for developing risk reduction strategies that target the needs of specific groups (Paton & Johnston 2001).

Meanwhile, resilient communities tend to withstand and recover better from disasters (Ashmawy 2021). As such, building resilience is promoted in the literature as a plausible approach to confront the increasingly devastating impacts of disasters (Aldunce et al. 2016). As Keating (2020:169–190) opines, the growing risks of disasters require the urgent need to enhance resilience. Since its inception in physics and material science (see Gößling-Reisemann, Hellige & Thier 2018), the meaning of the concept of resilience has continued to evolve and has gained prominence across many disciplines. Consequently, the concept is vastly contested (Berkes & Ross 2013) and does not have an internationally agreed-upon definition (Aksha & Emrich 2020). Despite its conceptual ambiguity, it is a valuable concept, and it has the potential to offer a more systematic and cross-cutting approach to DRR, climate change adaptation and the humanitarian sector (Aksha & Emrich 2020). Resilience refers to the capacities of the people, places and infrastructure to cope with hazards and involves long-term adjustments and learning processes to adapt to future events (eds. Fuchs & Thaler 2018). Norris et al. (2008) defined resilience as a process linking a set of adaptive capacities to a positive trajectory of functioning and adaptation after disturbance. They further argue that resilience is a process that leads to adaptation. Thus, developing resilience increases the community’s ability to thrive in dynamic environments that are characterised by unpredictability and surprises (Magis 2010).

With resilience as a process and not an outcome (Jiang, Ritchie & Verreynne 2021; Norris et al. 2008), building resilience should be an iterative and ongoing process (Linnenluecke 2017; Steiner & Atterton 2014). According to the National Research Council (2011), building and maintaining disaster resilience depend on the ability of a community to monitor change and then modify plans and activities appropriately to accommodate the observed change. Communities must reduce risks as well as resource inequalities, engage local people in mitigation, create organisational linkages, boost and protect social support, and plan for building community resilience (Norris et al. 2008). Building and strengthening communities’ resilience against disasters are some of the prominent aspects addressed in the goal of the Sendai Framework on Disaster Risk Reduction (SFDRR). According to Ashmawy (2021), resilient communities that have access to needed information and physical, economic, social and human capitals tend to prepare for and recover better from disasters.

The National Research Council (2011) refers to community resilience as the continued ability of a community to function during and after stress. Cutter et al. (2013) define community resilience as the ability to prepare and plan for, absorb, recover from and more successfully adapt to actual or potential adverse events. For Norris et al. (2008), community resilience is a process linking a network of adaptive capacities (i.e. resources with dynamic attributes) to adaptation after disturbance or adversity. The congruence between the different definitions of community resilience is that communities must possess certain capacities that must lead to adaptation. In this regard, four primary sets of adaptive capacities are essential for building community resilience, that is (1) economic development (level of economic resources, degree of quality in the distribution of resources and the scale of diversity in economic resources), (2) social capital (social support, social participation and community bonds), (3) information and communication (systems and infrastructure for informing communities, communication and narratives) and (4) community competence (collective action and decision-making, collective efficacy and empowerment) (Chu & Yang 2020; Norris et al. 2008; Sherrieb, Norris & Galea 2010). These adaptive capacities provide a roadmap for enhancing community resilience to disasters (Norris et al. 2008).

Berkes and Ross (2013) identify people–place connections; values and beliefs; knowledge, skills and learning; social networks; engaged governance (involving collaborative institutions); a diverse and innovative economy; community infrastructure; leadership; and a positive outlook, including readiness to accept change as a set of characteristics playing a pivotal role in building community resilience. Thus, community resilience means that communities can draw upon internal resources and competencies to manage demands, challenges and changes encountered (Paton & Johnston 2001). Engaging in activities for building community resilience instead of helping after a disaster will enable communities to fully leverage the resources and capacities of communities (National Research Council 2011). As Kanji and Agrawal (2020) argue, the primary need is to curb the focus on relief and rehabilitation, and emphasise preparedness, mitigation and resilience. The importance of building community resilience lies in that resilient communities can withstand hazards, continue to operate under stress, adapt to adversity and recover functionality after a crisis (National Research Council 2011).

As a result of the multifaceted nature of disaster resilience, any efforts to leverage the term to arrest the underlying drivers of increasing risk must be based on cooperation between stakeholders with complementary skills (Keating 2020). According to Cutter et al. (2013), there is a sustained need for coalitions at the local level to involve the whole fabric of the community in building resilience for collaborative problem-solving. As the National Research Council (2011) notes, the responsibility for building community resilience cannot and should not rest with the public sector alone. This is mainly because the government lacks leadership continuity, is characterised by bureaucratic rigidity, deficiency of financial resources and difficulty in innovating and transforming (Ashmawy 2021). This means that all sectors must collaborate to build community-level disaster resilience. As the National Research Council (2011) puts it, the collaboration between the private and public sectors and civil society organisation is vital because it helps improve the ability of a community to prepare for, respond to and recover from disasters. The SFDRR advocates for collaboration between the public and private sectors, civil society organisations, academia and scientific research institutions when reducing the risks of disasters, and for businesses to integrate disaster risk into their management practices (UNISDR 2015).

Therefore, creating and sustaining community resilience rely on the shared understanding that collaboration is beneficial for the entire community. Collaborative arrangements emerge when critical public and private sector actors recognise that individual and community goals cannot be effectively achieved through independent efforts alone (National Research Council 2011). The collective efforts of the public, private and civil society to identify in advance the interdependencies, needs and resources can significantly improve a community’s resilience to disasters (National Research Council 2011). Specifically, the private sector is the perfect advocate for resilience thinking because of its direct relationship with consumers, customers and suppliers and can steer public demand towards risk-sensitive products and services. In this regard, private sector organisations must choose to engage community resources in a way that brings benefit to local communities (Steiner & Atterton 2014). Watanabe (2009) encourages companies to work with local communities, including local residents and neighbourhood associations, chambers of commerce, and local governments and agencies. This is because communities can change many of the conditions that can increase their resilience (Berkes & Ross 2013). According to Aldunce et al. (2016), individuals and communities are key actors for building resilience, and therefore, there is an increasing need for their participation. Moreover, local communities constitute the first line of defence in reducing vulnerability and building resilience (Gaillard 2010).

On the other hand, businesses are vital centres of power and decision-making and that the actions of these firms touch the lives of citizens at many points (Kanji & Agrawal 2020). Private sector firms participate in community resilience-building initiatives because they have a vested interest in the area because of physical assets, suppliers, customers and corporate value of social responsibility (Stewart, Kolluru & Smith 2009). For Hamann et al. (2020), corporate managers worry about community resilience because they recognise their value chain interdependence with these communities and the associated socio-ecological systems upon which their business depends. Private organisations foster business continuity by offering resources and employment opportunities to the people and integrating social and ecological concerns in their business operations and corporate social responsibility (CSR) activities (Ashmawy 2021). Corporate social investment is a vehicle for voluntary advocacy and awareness-raising and funding support and the contribution of volunteers and expertise to implement risk management measures (Lal et al. 2012). Through CSR initiatives, companies should see DRR as an increasingly important development and humanitarian issue (UNISDR 2008). This is so because CSR provides a sound basis for encouraging businesses to participate in DRR and resilience building (La Trobe & Faleiro 2007). However, Van Niekerk et al. (2015) suggest that companies should start moving towards embracing the creation of DRM departments in their entities. As a starting point where a business is not involved in DRR, the government must invite private companies to become members of national platforms (eds. Izumi & Shaw 2015).

Watson et al. (2015) suggest that the private sector must invest in risk analysis and assessments, development of early warning systems, cost–benefit analysis and support to national risk reduction initiatives. Lal et al. (2012) identified three avenues for private sector engagement in DRR and resilience building: CSR, public–private partnerships (PPP) and business model approaches. Moreover, the major contributions of the private sector in DRR must be in the form of resources, expertise and capacities (eds. Izumi & Shaw 2015). According to UNISDR (2008), the private sector can play an important role in disaster prevention, mitigation and preparedness by investing more in DRR for business continuity and the local communities where their workforce resides. Van Niekerk et al. (2015) indicate that a private sector committed to DRR can steer public demands towards materials, systems and technological solutions to build and run resilient communities. Whether they perform these roles firm-centric (profitability, wealth creation and competitive corporate performance) or community-centric (helping and supporting the community and thus enhancing social and human capitals) (Ashmawy 2021; McKnight & Linnenluecke 2016), companies that actively engage in efforts to build community resilience may enjoy greater acknowledgement and standing in the community (National Research Council 2011). As Gaillard (2010) and Berkes and Ross (2013) assert, what is essential is that every effort to build community resilience must be sensitive to the unique social, cultural, economic, political and physical realities in which communities are embedded, thus making resilience context-specific.

## Research context

The research was conducted in Diepsloot, a community in the Johannesburg Metropolitan Municipality, South Africa. Diepsloot is a township situated north of Johannesburg and lies in the periphery of the most affluent suburban areas such as Dainfern, Fourways, Northgate and Sunninghill. Its development is as a result of people relocating to Diepsloot from Zevenfontein, and the banks of the Jukskei River in Alexander between 1991 and 2001, with the aim of government providing formal housing to the people (Harber 2011; Ngie 2012). However, this planned settlement was disrupted by the rapid influx of people into the area, in search of employment and to address the housing challenges (Himlin, Engel & Mathoho 2014). These new immigrants did not qualify for government subsidised housing units, which forced them to erect their own houses using corrugated iron (shacks) along what has been noted to be flood lines (Harber 2011; Ngie 2012). Brunsdon (2020:2) describes ‘shacks’ ‘as a unique African term that refers to dwellings constructed of unconventional building materials, such as corrugated iron, wood, cardboard and other materials’.

One of Diepsloot’s distinctive traits is its relative proximity to economic hubs attracting mainly people of employable age (Ngwenya & Zikhali 2014). The average age of the population in the area is reported to be 25 years (Ngwenya & Zikhali 2014). Diepsloot shows an unemployment rate of 30.2% (Ngwenya & Zikhali 2014). While 73.7% of the residents are considered to be economically active, only 47.0% are considered employed in mostly elementary or blue-collar jobs, including craft and related trades, service work, shop and market sales, and machine assembly (Johannesburg Development Agency 2012). The gender composition in Diepsloot is fairly balanced and shows a racial distribution of 97.0% black people and 23.0% shared amongst other races (Ngwenya & Zikhali 2014).

As a result of the rapid growth of informal settlements, Diepsloot is characterised as an area that is vulnerable to several urban hazards, including biological, chemical and physical hazards (Himlin et al. 2014). These hazards include potential structural fires, flash flooding and electrical hazards because of illegal electrical connections. This is a result of the area’s disparate socio-economic and environmental deficiencies, such as poverty, densely populated shacks and overcrowding. Furthermore, these factors have a profound bearing on the way in which the community and its local municipality reduce risks and build resilience. Substantially evident in the area is a robust and highly progressive private sector, residential and commercial development which include estate housing developments, corporate business parks and shopping centres.

## Research methods

The study draws upon a range of data collection techniques including a thorough literature review on the role of the private sector in building disaster resilience, focus group discussion (FGD) and face-to-face interviews. A phenomenological approach, which is associated with qualitative design, was applied to the study. Phenomenological approach focuses on the ways in which individuals make sense of their social worlds (Bryman 2008). As such, a researcher must understand the social phenomena as experienced by the individuals in the study and present the data as conveyed by the individuals being studied (Bryman 2008; Trochim 2006). A total of 50 community members and five private companies’ representatives participated in the study.

All respondents were selected purposefully as they were deemed to meet the salient features of the study (Strydom & Delport 2005). Data were collected through FGD with community members and face-to-face interviews with the CSR managers of the selected private companies in the vicinity of Diepsloot. All interview responses were transcribed verbatim and initially checked and categorised manually to identify core ‘presenting’ themes and patterns. Data were analysed thematically following the guidelines provided by De Vos et al. (2011). Thematic analysis is a categorising approach in which data are categorised into themes and sub-themes identified during the data collection process (Bryman 2008).

## Findings and discussion

The study sought to explore collaboration between private companies and the community of Diepsloot to build disaster resilience of the community. The findings of the study are presented according to the themes that emerged during the interviews and these provide a comprehensive picture of the views and experiences of the respondents. The findings echo what many scholars emphasise in literature, that through their CSR programmes, the private sector is in a good position to build community resilience by addressing the factors that make communities vulnerable to disasters.

### Nature of private company engagement in Diepsloot

The findings of the interviews with representatives of private companies reveal that several companies are involved in initiatives that are aimed at addressing the socio-economic challenges faced by communities in Diepsloot. All the respondents from the private companies indicated that they had engaged with the community of Diepsloot through their CSR programmes or projects. The findings reveal that these initiatives took place by either collaborating with a Community Based Organisation (CBO) already existing in the community or had a non-profit section within the company that establishes a structure within the community to implement the initiatives. Through partnerships with the local CBOs, the nature of each company’s engagement differ and has either direct ties to their day-to-day function or in other cases have nothing to do with their function. Table 1 provides an overview of the type of community engagement projects undertaken by private companies in Diepsloot as outlined by the respondents from the private companies.

**TABLE 1:** Community engagement projects in Diepsloot.

Respondent	Internet services	Property development	Tourism	Insurance	Health
Types of community engagement projects	Skills development	Residential care for children orphaned by AIDS	Construction of a library	Skills development	Early childhood development (ECD) support
	Free Wi-Fi	Construction of church/multi-purpose centre	Providing library resources	Job placement	-
	School support	Early childhood development (ECD) support	Reconstruction of temporary shelter	-	-
	Employee engagement	Cleaning up of the Jukskei River	Donation of blankets and stationery	-	-

The respondents from the property development company indicated that their CSR activities were implemented with the assistance of their own founded CBO. The respondents indicated that rather than working through already established structures within the community, they establish their own structure to assist with the implementation of their initiatives. The respondents further indicated that, in Diepsloot, they have built a children’s village that provides residential care to children orphaned by acquired immunodeficiency syndrome (AIDS). In addition, the respondents indicated that they also constructed a multi-purpose centre that the community utilises for church services; training and youth development workshops; and counselling services. Further to this, they also assisted the community in cleaning up a section of the Jukskei River that runs through Diepsloot. The company focusing on tourism built a library in Diepsloot as part of their CSR initiatives. They further sponsored the resources to be used in the library; the reconstruction of low-cost housing for residents in the community; donated blankets and stationery to the community; and encouraged employee engagement in these initiatives.

The company working in the insurance industry provided skills development and recruitment solutions in order to address unemployment and to reduce poverty levels, thus improving the lives of people living in Diepsloot. A company representative from the insurance industry indicated that they have focused on capacity building, training, recruitment and selection of unemployed youth in Diepsloot. The respondents indicated that their activities are done in collaboration with one of the youth development agencies in the area. This respondent stated:

‘Our CSR [*corporate social responsibility*] strategy is to improve the lives of people living in Diepsloot through the skills development and recruitment programmes we have implemented together with a youth development agency in Diepsloot.’ (Respondent 1, CSR manager, insurance industry)

Contrary to the positive responses from private company representatives regarding their involvement in contributing to the improvement of the lives of people living in Diepsloot, local residents who participated in the FGDs held different views. The majority of the focus group respondents indicated that, whereas they were aware of companies that operated in close proximity to the community of Diepsloot, they were not aware of any community projects by the private companies in the area. The few respondents who indicated that they are aware of private company activity were able to identify a real estate company that was involved in the community. The company was not part of the companies that participated in the study. It is alarming that none of the companies that participated in the study, which claimed to have initiatives in the community, were identified by the community respondents from the study. This might be due to the fact that their initiatives are implemented in areas that were not represented in the study.

Respondents from the FGDs who were aware of the private companies’ initiatives were of the view that private company engagement in the community was firstly based on an assumption of the community’s needs. Secondly, that private company initiatives were centred on certain sections in Diepsloot that are more developed. The essence of this is highlighted in the statement below made during FGD:

‘When these private companies do their research, they must do it all over Diepsloot and not just the sections in Diepsloot that are well-developed.’ (Respondent 2, community member, Diepsloot)

Such responses suggest that not all community-based interventions by private companies reach the entire community. Therefore, improved collaborative measures are required to address the visible gap between CSR operations and community engagement.

### Community consultation for effective collaborative partnerships

All the respondents from the private companies were of the firm view that it is important for private companies to assist in addressing the challenges that the communities in their vicinity face. For example, one respondent from the private companies cited the challenging task the government has to address the socio-economic needs of the country. They also pointed out that it is important for private companies to assist government to address these challenges. It is well documented that socio-economic factors are the underlying issues that render communities vulnerable to disasters and exposed to hazards. Thus, addressing socio-economic challenges that communities face is important for reducing the risk of disasters and in building a community’s disaster resilience. One respondent stated:

‘We recognise the socio-economic challenges in the community and the burdensome role the Government has in responding to these challenges. As a business, we respond to the needs that echo our core business strategic areas.’ (Respondent 4, CSR manager, Internet services)

Both private company representatives and members of the community concurred that it is important for private companies to consult with the communities before implementing any initiative. The statement below captures the essence of this:

‘From my experience in working with various impoverished communities, the basis of any successful community project is the consultation with the communities before any project is implemented. By doing this, you get the community’s buy-in and also have a fair idea of the needs of the community. In this way, the company is able to contribute in a meaningful way.’ (Respondent 5, CSR manager, property development company)

The importance of consultation with organised community structures was reflected across all the responses from the private company interviews. Private company respondents indicated that they rely on CBOs and schools within the community to implement their initiatives. This is because private companies consider CBOs to have more knowledge about the community and ways to intervene and an understanding of various social challenges eminent in the community. This finding converges with the literature. Gil-Rivas and Kilmer (2016) are of the view that partnering with an area’s residents and stakeholders, recognising their strengths and assets and appreciating their local knowledge and lived experiences is crucial for disaster preparedness and response. UNISDR (2007) points out that adopting participatory approaches to problems and solution identification creates self-confidence amongst the poorest and the most vulnerable families.

While some FGD respondents felt that schools or CBOs provided a link between the community and the private companies, some felt that private companies needed to approach the community directly. These respondents felt that community interventions by CBOs and schools were inaccessible. They further argue that the use of schools and CBOs is the reason why some sectors of the community are not aware of the initiatives by private companies. Implementation of projects through CBOs and schools that are unknown to the wider community might cause the project to be perceived as unimpactful, ineffective and exlusionary of certain people. The statement below captures the essence of those who advocate for private companies to approach the community directly:

‘It is important that companies build a relationship with the community members and not just the school or one section of the community.’ (Respondent 3, community member, Diepsloot)

Such assertions suggest that CBOs used by private companies in community engagement, including the schools in the community, did not represent all the members of the community.

### The importance of organised community structures for collaboration

As reflected in the section above, all private company respondents indicated that they prefer to work with CBOs and schools as implementers of their CSR programmes. While some companies establish their own community structures, others prefer using structures that are already in existence in the community such as CBOs and schools. As justification for their preference to work with organised community structures, private company respondents cited their historical experiences of working directly with the community members. For these respondents, it takes a lot of effort to manage the relationship between the company and the community members in terms of time and resources. Moreover, private company respondents argued that it is easier to have someone specific accountable for how the resources were used when working with organised structures in the community.

While contributing to the well-being of the community is the objective of the private companies’ CSR mandates, findings reveal that private companies choose carefully the types of CBOs they work with. Some of the private company respondents referred to the importance of reputation and social standing of the CBOs they consider as a partner to collaborate with. These respondents indicated that the status and reputation of the organisation within society are important.

It is, however, important to note that the views of the focus group respondents do not disregard the contributions made by private companies towards addressing the vulnerabilities identified in the community regardless of the means of implementation. Interestingly, majority of respondents from both private companies and local residents that participated in the study recognise the need to have organised structures through which community engagement projects by private companies can be implemented.

### Building the capacity of communities to respond to adverse events

Enhancing the capacity of communities to respond to disaster occurrences form the basis for reducing disaster impacts, minimising the effects of hazards and building disaster resilience. The UN (2016) describes capacity as the combination of all the strengths, attributes and resources available within an organisation, community or society to manage and reduce disaster risks and strengthen resilience. This may include infrastructure, institutions, human knowledge and skills, and collective attributes such as social relationships, leadership and management. Gil-Rivas and Kilmer (2016) are of the view that human and social aspects of communities are key resources in the face of disaster. In addition, community resilience includes mainly community-level factors that protect the community from adverse experiences as opposed to the factors within an individual that determines his/her resilience. This implies that for the community to build its resilience, it will be imperative for collaboration with the private companies to be directed towards enhancing the capacity of the community to respond to and cope with disaster events. As Paton (2006) posits, that the capacity for adaptation and growth exits within the community should not be taken to imply that communities must just be left to fend for themselves. This means that those in the position to assist communities (in this study, private companies) should build the capacity to respond to disaster and should help the communities.

#### Availability of resources

Both focus group respondents and private company representatives concur that private companies have contributed towards addressing the socio-economic challenges that the community face. It can thus be argued that in the process of addressing socio-economic challenges, private companies contribute to addressing the factors that make communities vulnerable to disasters. Therefore, contributing to building resilience. These findings suggest that private companies may not be consciously aware of the contribution made by their CSR programmes towards addressing the vulnerabilities and disaster risks eminent in the community. It has also emerged in this study that private companies provide the communities with necessary resources such as employment, skills, access to social services, and housing. Such resources are valuable to building the community’s resilience. For example, company representatives from the property development company indicated that they have constructed a multi-purpose centre for various community-related services, events and training. This centre contributes directly to strengthening the social capital of the local residents. Furthermore, the company representative in the health services indicated that the company donated shipping containers for extra classroom space. Moreover, the private company in the information and communication technology (ICT) services has created a number of job opportunities. One of the respondents in the ICT sector stated:

‘Our CSR strategy is to improve the lives of people living in Diepsloot through the skills development and recruitment programmes we have implemented together with a youth development agency in Diepsloot.’ (Respondent 1, CSR manager, insurance industry)

An important aspect that was mentioned in the interviews with the private companies was the importance of financial resources to fund community projects. The majority of the private company respondents indicated that they have limited financial resources to work with. As a result, they continually try to find alternative and innovative ways to conduct their community engagement projects in order to create value for both the community and the company. Such mechanisms include prioritising corporate spending based on a needs analysis, committing their time towards community engagement activities instead of providing direct financial donations and donating resources such as shipping containers in support of the Early Childhood Development (ECD) Centre in Diepsloot or library resources after constructing the library in the community. This shows that private companies that participated in this study are willing to assist and address community challenges considering minimal financial availability. To address the lack of funding, private companies resort to corporate fundraising in order to support their projects. One respondent stated:

‘This project [*residential care facility*] requires huge amounts of money. As a company, we are unable to do it alone hence the foundation and the CBO have approached other companies to assist.’ (Respondent 5, CSR manager, property development company)

Respondents from the community who participated in FGDs were of the view that private companies have the resources to assist communities in addressing vulnerabilities that may lead to disaster risks within the community.

‘Well, for me, I think private companies can teach us about fires and how to prevent them and how to stop the fires when they happen.’ (Respondent 6, community member, Diepsloot)

Responses show that financial resources play an important role in community engagement. This, however, does not mean that the companies will not engage with communities in the absence of financial resources. From the findings of the study, it is evident that private companies are willing to assist and are eager to find alternative ways in which they can address the challenges that the communities face. What was commonly expressed by these respondents was the importance of their community projects having sustainable and valuable outcomes for both the business and the community. As Gil-Rivas and Kilmer (2016) indicated, the strengths of resources to address the communities’ needs depend on their number and diversity, and the ways they are distributed in the society. This brings us to the concept of social capital, which is discussed in the next section.

#### Enhancing collective action through social capital

According to Putnam (1993), social capital refers to the existence of organisational networks that have reciprocal, supportive and trusting relationships; have moderate levels of overlap with other networks; are able to form new associations with other social networks; and have the ability to make joint decisions in a collaborative manner. In this study, there was a general awareness of the existence of social groups in the community across the majority of the focus group respondents. Most of the focus group respondents reported on the presence of social groups and acknowledged that the social groups are formed in order to supplement household resources. While some focus group respondents indicated that they are not part of any social group, some respondents said that they had membership in various social groups, such as church-based groups, church-based social clubs, youth groups and stokvels for groceries and funerals:

‘We do have social groups in the community. For example, I am part of a stokvel where we help with contributions at funerals.’ (Respondent 8, community member, Diepsloot)

The common perspective across the focus groups respondents was that social groups were not formed for disaster-related activities. This being the case, social ties can be used for other purposes beyond what they were established for and therefore members of the network can turn to their network for assistance in times of crisis. The findings from FGDs revealed that the assistance of community members during difficult times was not only dependent on the social groups but also from the social networks, community vigilantes or the Community Protection Forums (CPFs). The willingness and ability to assist during difficult times reinforce the importance of collective action and thus the level of social capital within the community. Contributing to this point, resources provided as in the example given before about the multi-purpose centre, gives social groups a platform to work from and organise around. Private companies therefore do not dictate or influence the workings of the active social groups in the area but support them in providing a facility. Gil-Rivas and Kilmer (2016) are of the view that formal and informal social ties are of relevance because they facilitate access to economic and informational resources.

Moreover, the focus group respondents indicated that the extent to which community members participate in the social groups depends on the reason for the group’s existence. For example, it was indicated that the participation of community members in a particular social group was dependent on the returns/benefits obtained from the social group. This finding indicates that community members based the value of their participation in the social groups on the returns expected from these social groups.

‘Community members are likely to join a stokvel than joining a church group because stokvels bring money.’ (Respondent 7, community member, Diepsloot)

Such assertions are in line with the literature. For instance, the literature refers to membership of a social network as a personal sense of belonging to and investment in the community because it confers to individuals a sense of emotional safety and identification with the community (McMillan & Chavis 1986).

#### Community competence in addressing adverse events

According to Cottrell (1976 as cited in Gil-Rivas & Kilmer 2016), the capacity to collaborate effectively, agreeing on goals, priorities and strategies for achieving them, and engaging in collective action indicates a community’s competence. Community competence largely depends on social capital and communication, which are resources that can enhance the community’s capacity to reach joint decisions and take collective actions to prepare for, respond to and cope with disasters (Gil-Rivas & Kilmer 2016). In this study, the findings from focus group interviews revealed that there is a high level of community competence. Respondents highlighted the community’s collective ability to mobilise community members in order to respond to a disaster occurrence. Even when local residents feel under-resourced, they still act. An example of a shack fire was given. One respondent remarked:

‘We as the community are ready and willing to help our neighbours when there is a shack fire but at times, we don’t have the resources to do so.’ (Respondent 9, community member, Diepsloot)

Although data from the focus groups showed that CPFs and private companies do not necessarily prioritise on activities for DRR, it revealed that the presence of private companies and CPFs may serve as an important capacity where DRR-related activities can be discussed and addressed. This will help in enhancing the competence of the community to plan for and respond to disasters.

### Disaster risk perception and knowledge

Ardaya, Evers and Ribbe (2017) view disaster risk perception (DRP) as a predecessor of mitigation behaviour. Mitigation means lessening or minimising of the adverse impacts of a hazardous event (UN 2016). In this study, the perception of the focus group respondents about crime was much more firm than their perception on disasters because of the prevalence of crime in the community. This is evidenced by the establishment of CPFs and the vigilante groups who intervene on issues of violent crime. To the contrary, the community does not have an organised group of disaster volunteers. Despite this situation, the majority of the focus group respondents expressed a high-level knowledge about disasters frequently occurring in the area over the past 5 to 10 years. Respondents identified the most frequently occurring events to include shack fires resulting from illegal electrical connections, paraffin stoves and candles; flooding and drowning of school children in the Jukskei River, violent crimes and health-related hazards such as rat infestations due to waste pollution.

With the knowledge that the majority of focus group respondents have on past disaster events and prevalent hazards in the area, they expressed their willingness to work with private companies in order to address the hazards that led to the disaster. The majority of respondents referred to shack fires that have occurred in the community and as an example of hazards private companies can assist in addressing:

‘Well, for me, I think private companies can teach us about fires and how to prevent them and how to stop the fires when they happen.’ (Respondent 10, community member, Diepsloot)

The knowledge and perception of disaster risk in the community are important to help the community address the risk. As Ardaya et al. (2017) posit, the value of risk appraisal and perception lies in that they help modify risk management decision and management actions.

### The importance of creating awareness on disaster risk and addressing communities’ vulnerabilities

Whereas the community has experienced several disasters in the past, the findings from the FGDs reveal that the community is not well-informed about disaster risk and the measures to reduce such risk. The majority of the focus group respondents acknowledged the lack of education and knowledge in relation to disaster risks, reduction and management. The statement below captures the views of the focus group respondents on the issue of disaster risk education.

‘I don’t think the community is educated about disasters, we are not.’ (Respondent 11, community member, Diepsloot)

Besides, findings from the FGDs reveal that some community members hold the perception that there are people within the community who are well informed about DRR. One respondent stated:

‘… but I feel that there are members within the community that have the knowledge but are not sharing it, especially those that are well educated and have good jobs.’ (Respondent 12, community member, Diepsloot)

On a similar note, data from the private company interviews revealed lack of understanding on issues relating to DRR. The majority of private company respondents linked their community engagement projects to their overall business objectives and therefore do not consciously prioritise DRR in their CSR projects in the community. The inability of private companies to make the connection between their community engagement projects and the DRR suggests that private companies are not well-informed about the role they must play in reducing the risk of disasters.

Findings from both FGDs and private sector respondents suggest that there is a need to create awareness on disaster risk and ways of addressing the vulnerabilities in the community that result in disaster risk events. The private sector must start to safeguard business continuity in the face of disasters. They must also be driven by the desire to assist the communities likely to be affected by disasters. To better understand their role and responsibilities in DRR, companies must start to participate in government-led multi-stakeholder forums.

## Conclusion

The findings of this study showed that private companies in the vicinity of the study area are involved in initiatives to address the socio-economic challenges of the community. After all, socio-economic factors are amongst the factors that lead to communities being vulnerable to hazards. Socio-economic factors also limit the communities’ coping capacity and thus reduce the community’s resilience to disasters. Generally, respondents understood the importance of private companies to address the factors that could increase the community’s resilience to disasters. The findings of this study revealed that the private companies have the potential to serve as an important mechanism through which the capacity of the community to respond to adverse events can be enhanced. Furthermore, the findings reveal that, while some sectors of the community prefer for companies to work directly with the communities, some respondents from the community and those representing private companies prefer to engage the communities through community structures. Those against the use of community structures cite the inaccessibility of the structures by the entire community, while those in favour cite manageability and accountability as important. In line with those who are in favour, this study recommends the use of community structures when private companies engage in community initiatives. Moreover, the study recommends that the South African Government must be proactive in making the business case for private companies to get involved in disaster preparedness, response, recovery and building back. The limitation of this study is that with the majority of private companies within the vicinity of the study area not participating in the study, the findings cannot be generalised to represent the situation in the entire country. With this study focusing on small micro-enterprises, future research can thus focus on big companies such as multinational and national companies in South Africa.
